# Reaching Staff, Parents, and Community Partners to Prevent Childhood Obesity in Head Start, 2008

**Published:** 2010-04-15

**Authors:** Rachel A. Gooze, Cayce C. Hughes, Daniel M. Finkelstein, Robert C. Whitaker

**Affiliations:** Temple University, Philadelphia, Pennsylvania; Temple University, Philadelphia, Pennsylvania; Mathematica Policy Research, Inc, Cambridge, Massachusetts; Temple University, Center for Obesity Research and Education

## Abstract

**Introduction:**

Lowering the prevalence of childhood obesity requires a multilevel approach that targets the home, school, and community. Head Start, the largest federally funded early childhood education program in the United States, reaches nearly 1 million low-income children, and it provides an ideal opportunity for implementing such an approach. Our objective was to describe obesity prevention activities in Head Start that are directed at staff, parents, and community partners.

**Methods:**

We mailed a survey in 2008 to all 1,810 Head Start programs in the United States.

**Results:**

Among the 1,583 (87%) responding programs, 60% held workshops to train new staff about children's feeding and 63% held workshops to train new staff about children's gross motor activity. Parent workshops on preparing or shopping for healthy foods were offered by 84% of programs and on encouraging children's gross motor activity by 43% of programs. Ninety-seven percent of programs reported having at least 1 community partnership to encourage children's healthy eating, and 75% reported at least 1 to encourage children's gross motor activity.

**Conclusion:**

Head Start programs reported using a multilevel approach to childhood obesity prevention that included staff, parents, and community partners. More information is needed about the content and effectiveness of these efforts.

## Introduction

Lowering the prevalence of childhood obesity requires a coordinated, multilevel approach that goes beyond the home to target schools and communities (1). However, there are few successful examples of implementing such a multilevel approach (2,3), and we are not aware of any that have been evaluated in early childhood, when obesity prevention efforts should begin (4).

Head Start, the nation's largest federally funded early childhood education program, presents a unique opportunity to implement a multilevel approach to prevent childhood obesity in a population at high risk for obesity. Head Start reaches nearly 1 million low-income preschool children. It uses an approach to school readiness that integrates children's cognitive, social, and emotional development with their physical health and that emphasizes the need for staff training, parent involvement, and community partnerships (5,6). This approach, used from the inception of Head Start, was informed by Bronfenbrenner's ecological theory of human development (7), which accounts not only for the multiple levels of influence on the child but also the need for synergy between the school, home, and neighborhood environments. The social ecological model has been widely applied in public health (8,9).

All Head Start programs must abide by regulations outlined in the federal Program Performance Standards (10), which include those that apply to staff training, parent outreach, and community partnerships, providing an administrative structure for implementing a multilevel approach to prevent obesity. For example, the regulations require Head Start programs to provide ongoing training for their staff (10), to hire staff or consultants to support family and community partnerships, to "provide health and nutrition education for parents and families," and to "take affirmative steps to establish ongoing collaborative relationships with community organizations." Programs must establish parent committees and convene a health services advisory committee.

Despite an existing structure in Head Start to allow for the development of a multilevel approach to preventing childhood obesity and the need for such an approach (11,12), no national data are available to indicate how Head Start programs are encouraging healthy eating and physical (gross motor) activity in children through activities directed at staff, parents, and community partners. The adults reached through these activities can model healthy behaviors and implement the obesity prevention practices that are intended to target children. Using data collected in a 2008 national survey of Head Start programs, we describe obesity prevention activities directed at staff, parents or guardians, and community partners.

## Methods

The Office of Head Start in the US Department of Health and Human Services (DHHS) administers grants, through 12 regional offices, to almost 1,900 Head Start programs. These programs use the grant funds to administer services to almost 1 million low-income preschool children in 50 states, the District of Columbia, and US territories (13). The average Head Start program has approximately 6 centers, each with 50 to 60 children aged 3 or 4 years.

From February through April 2008, we administered a survey to all Head Start programs as part of the Study of Healthy Activity and Eating Practices and Environments in Head Start (SHAPES). The purpose of the survey was to provide the first national description of obesity prevention practices and environments in Head Start, focusing on both healthy eating and gross motor activity. The surveys were addressed to program directors, who were encouraged to get assistance with the survey from their program's specialists in health or nutrition.

### Survey development and administration

The survey instrument was developed and administered in partnership with DHHS and the US Department of Agriculture (USDA), which supplies meals and snacks to Head Start through the Child and Adult Care Food Program (14). Drafts of the instrument were also reviewed by several nonfederal content experts, and it was further refined on the basis of cognitive interviews and pretesting with 7 Head Start program directors, each from a different state. The final survey did not require program staff to conduct any record review and could be completed in approximately 30 minutes. To reduce bias, we assured programs that their individual responses would not be shared with federal agencies. The term "gross motor activity" was used instead of "physical activity" because it was more familiar to Head Start staff.

Administrative data and contact information for all 1,890 Head Start programs were obtained from the Office of Head Start's 2007 Program Information Report (15). We excluded 50 programs in US territories, 27 that did not provide direct services to children, and 3 that provided all services outside of centers, leaving a final sample of 1,810 programs. Program directors were mailed a paper survey. After sending reminders by electronic and postal-service mail, we reached nonresponding programs by telephone and allowed them to complete the survey over the telephone.

### Survey items

This report focuses on responses to closed-ended survey questions about activities in Head Start programs that were directed at adults — staff, parents or guardians, and community partners — rather than at the children. To understand the perceptions of program directors about the magnitude of the problem of obesity in their program, we asked the following question: "In your opinion, how much of a health problem is obesity among the *children* in your program?" The response options were "not a problem at all," "a small problem," "a moderate problem," "a large problem," or "a very large problem." In 2 other similarly worded questions, we asked about obesity among *staff* and among *parents*.

We asked programs how they trained newly hired staff about practices and routines that apply to feeding children at snacks and mealtimes. Programs were given a list of training practices and asked to mark all that applied. They were then asked to indicate the most commonly used practice on the list. A similar pair of questions was asked about training practices that apply to children's gross motor activity. In addition, we asked (yes/no) whether programs offered workshops or activities for staff members to assist them with improving their own eating and physical activity behaviors.

From a list of activities, we asked programs to indicate which ones they used during the past year to encourage parents or guardians to provide opportunities for children's healthy eating at home. A similarly worded question asked about opportunities for gross motor activity at home. For both of these questions, programs were asked to mark all that applied from a list of activities and were given the opportunity to write about other parent outreach activities that were not on the list. In addition, programs were asked (yes/no) whether they provided opportunities for parents or guardians to participate in menu planning for foods and beverages that are served at the program. Finally, programs were asked (yes/no) whether, during the past year, they had involved their parent committee as part of any efforts to prevent obesity among young children.

From a list, programs were asked to indicate the types of community organizations and agencies with which they had partnerships during the past year to encourage children's healthy eating and, in a separate question, to encourage children's gross motor activity. Finally, programs were asked 2 (yes/no) questions: whether, during the past year as part of any efforts to prevent obesity among young children, they had 1) formed a *new* partnership with a community organization, and 2) involved their health services advisory committee.

### Data analysis

We described the percentage of programs reporting various activities with 1) staff, 2) parents or guardians, and 3) community partners. For the questions on parent outreach activities, we coded into subgroups those "other" activities that were written in by programs. There was no subgroup of activities that made up more than 5% of the total sample; therefore, these activities were not reported separately. In reporting results we used the term "parents" to refer to parents or guardians.

## Results

The 1,810 programs enrolled 828,707 children across 13,607 centers, 89% and 90% of all Head Start children and centers, respectively. Surveys were completed by 1,583 (87%) programs, 188 by telephone.

Forty-seven percent of program directors perceived that obesity was a large or very large problem for parents, 33% perceived that obesity was a large or very large problem for staff, and 20% perceived it was a large or very large problem for children.

### Activities with staff

Nearly all programs provided newly hired staff with some training about the practices and routines that applied to feeding children and to children's gross motor activity. Only 3% and 6% of programs reported no training of new staff (other than observing more experienced staff) on feeding and on gross motor activity, respectively (Table 1). Programs reported on their use of 4 methods to train new staff about feeding and gross motor activity: 1) having an experienced staff member verbally explain the practices and routines to new staff, 2) providing workshops or training sessions for new staff, 3) asking new staff to read materials, and 4) asking new staff to view videotapes. For training on feeding, 86% of programs used at least 1 of the 3 other methods besides verbal explanation, and 83% did so for training on gross motor activity. In addition, 50% of programs reported that they offered workshops or activities for staff members during the last year to help them improve their own eating and physical activity behaviors.

### Activities with parents

Distributing written information, such as flyers or newsletters, was the approach that programs most often reported using to reach parents about providing opportunities at home for children's healthy eating (Table 2). However, most programs went beyond distributing written materials. For example, 84% of programs offered a workshop for parents on either preparing or shopping for healthy foods, and 60% of programs reported that they discussed healthy eating at parent-teacher conferences. Beyond these approaches, 12% of programs reported other types of parent outreach on healthy eating, such as providing healthy meals and snacks at parent events or referring families to a physician, nurse, or nutritionist if a problem with nutrition or weight was identified. Only 4 programs (<1%) reported offering no parent outreach activities on healthy eating in the last year, 27% offered 1 or 2 types of activities, 32% offered 3 types, and 41% offered more than 3 types. In addition, 80% of programs reported that they provided opportunities for parents or guardians to participate in menu planning for foods and beverages served at Head Start meals, and 40% of programs reported involving their parent committee during the past year in their overall childhood obesity prevention efforts.

Distributing written information was also the most common approach to parent outreach about opportunities for children's gross motor activity at home (Table 2). Almost three-fourths of programs reported that they discussed children's gross motor activity at parent-teacher conferences, and 43% offered a workshop that taught parents how to encourage children's gross motor activity at home. Beyond these approaches, 6% reported other types of parent outreach on gross motor activity, such as discussing the topic with parents during home visits. Seven percent of programs reported no parent outreach activities in the last year on gross motor activity, 25% reported 1 type of activity, 38% reported 2 types, and 30% reported 3 or more types.

### Activities with community partners

The Special Supplemental Nutrition Program for Women, Infants, and Children (WIC) (16) was the most common community organization or agency with which Head Start programs had a partnership during the prior year to encourage children's healthy eating, followed by the USDA cooperative extension program and the local public health department (Table 3). Ninety-seven percent of programs reported having at least 1 community partnership to encourage children's healthy eating, 28% had 1 or 2, 23% had 3, and 46% had 4 or more.

WIC was also reported as the most common community organization or agency for partnerships to encourage children's gross motor activity, followed by the health department and a school or school district (Table 3). Seventy-five percent of programs reported having at least 1 community partnership to encourage children's gross motor activity, 27% had only 1, 23% had 2, and 25% had 3 or more.

As part of their overall efforts in the past year to prevent childhood obesity, 73% of programs reported involving their program's health services advisory committee. Nineteen percent formed a new partnership with a community organization or agency.

## Discussion

In this national survey, we found that Head Start programs reported using a multilevel approach to childhood obesity prevention, which included activities directed at staff, parents, and community partners. They offered workshops to parents about preparing and shopping for healthy foods, trained new staff on children's feeding and gross motor activity, provided activities for staff to improve their own eating and activity habits, and established partnerships with community organizations to help prevent childhood obesity. These activities can reach the salient adults in children's lives and establish positive and consistent social norms for children regarding diet and physical activity. The importance of reaching these adults is reflected in the fact that many Head Start program directors considered obesity to be a substantial problem for both parents and staff.

Many childhood obesity prevention efforts have taken place in schools, where children spend a great deal of time and where the environments related to both diet and physical activity can be altered (17). However, children consume most of their calories outside the school setting (18,19). Furthermore, seasonal patterns of weight gain in young children suggest that the nonschool environment may be more influential than the school environment (20). A more effective approach may be to reach children in the multiple contexts in which they spend their time, not only in school but also at home and in their neighborhoods. Few examples of such multilevel approaches to childhood obesity prevention have been evaluated (2,3), and their activities were centered in schools but did not specifically involve preschools or child care settings.

A major challenge in this school-centered approach is that the primary focus in elementary and secondary schools is on academic achievement. Additionally, these schools are not inherently oriented to an ecological model of child development, nor do they include children younger than 5 years in whom health habits are already being established. Applying a multilevel approach to obesity prevention in Head Start, however, has many advantages because Head Start reaches children at younger ages, integrates children's health, nutrition, and gross motor development, and requires involvement of staff, parents, and community partners. Additionally, the Program Performance Standards require that former or current Head Start parents be given preference for Head Start staff positions for which they are qualified (10). The fact that more than one-fourth of staff are former or current Head Start parents (21) means that efforts to reach parents about obesity prevention will also reach some future staff. In addition, Head Start could frame some of its messages about obesity prevention in a similar way for parents and staff.

Since its inception, Head Start has focused on children's health, recognizing the relationship between health and children's ability to learn (22). These efforts have involved staff, parents, and community partners. For example, in its recent initiatives to improve children's oral health, Head Start programs received grant support to build connections with dentists and dental hygienists in the community and to increase education of parents (23). Head Start has also applied an ecological approach to address children's mental health, using interventions that include both parent and staff training (24,25).

As with oral and mental health, effective and sustainable models for obesity prevention will likely require involvement of staff, parents, and community partners. A promising example of obesity prevention efforts in Head Start is the I Am Moving, I Am Learning initiative, a program enhancement designed to encourage children's moderate to vigorous physical activity, adult-guided movement activities, and healthy eating behaviors (26). Of the 50 programs participating in the early implementation of I Am Moving, I Am Learning, more than half offered activities for staff about their own diet and physical activity behaviors, nearly all provided activities for parents, and more than half formed a partnership with at least 1 community organization to prevent obesity (27).

Despite the high response rate to the SHAPES survey, which attempted to reach all Head Start programs, this study had several limitations. We did not validate program reports of their activities by conducting on-site interviews of staff, parents, or community partners. This was not an evaluation in which we tried to assess details about the implementation (content and intensity), reach (number of adults who participated), and effectiveness of the reported activities (28). In addition, the survey required programs to respond to questions on the basis of the average or typical Head Start center in their program. Programs with large between-center variability might have been more likely to misclassify their program's activities.

Considering that young children can benefit in many ways from links between the school, home, and community (29-31) and that there is growing interest in obesity prevention efforts in early childhood education settings (4,32), more information is needed on how early childhood programs are implementing such links in their obesity prevention efforts. The philosophical and administrative foundation for a multilevel approach to obesity prevention is already in place in Head Start. We now have national data on the types of staff training, parent outreach activities, and community partnerships used in Head Start to encourage children's healthy eating and gross motor activity. Future research is needed to explore the content and effectiveness of these strategies.

## Figures and Tables

**Table 1:** Staff Training Activities About Feeding Children and Children's Gross Motor Activity, US Head Start Programs, 2008 (N = 1,583)

Activity	% of Programs Offering Activity^a^
**Feeding children (n = 1,576)^b^**	
An experienced staff member verbally explains practices and routines that apply to feeding children^c^	92
Staff attend a workshop or training session about feeding children	60
Staff are asked to read books or articles about feeding children	14
Staff view videotapes about feeding children	13
No training for new staff about feeding children other than observing what the most experienced staff do during meals and snacks	3
**Gross motor activity (n = 1,574)^d^**	
Experienced staff member verbally explains practices and routines for encouraging children's gross motor activity^c^	87
Staff attend a workshop or training session about children's gross motor activity	63
Staff are asked to read books or articles about children's gross motor activity	25
Staff view videotapes about children's gross motor activity	18
No training for new staff about children's gross motor activity other than observing what the most experienced staff do during children's gross motor activities	6

a Percentages do not total 100 because programs were allowed to report more than 1 activity.

b Seven programs that did not respond to the question were excluded.

c In addition to reporting which of the listed activities were used, programs reported on which one was the most commonly used. For feeding, 69% of programs reported that the most common training activity was verbal explanations of the practices and routines; for gross motor activity, 66% of programs reported that this was the most common training activity.

d Nine programs that did not respond to the question were excluded.

**Table 2:** Activities for Encouraging Parents to Provide Opportunities for Children's Healthy Eating and Gross Motor Activity, US Head Start Programs, 2008 (N = 1,583)

Activity	% of Programs Offering Activity^a^
**Healthy eating (n = 1,579)^b^**	
Distributed written information (flyers, pamphlets, or newsletters) about healthy eating	97
Offered workshops or events that taught parents how to prepare healthy foods	80
Offered workshops or events that taught parents how to shop for healthy foods	64
Discussed healthy eating at parent-teacher conferences	60
Other	12
Did not conduct any activities	<1
**Gross motor activity (n = 1,572)^c^**	
Distributed written information (flyers, pamphlets, or newsletters) about opportunities and facilities in the community for children's gross motor activity	78
Discussed gross motor activity at parent-teacher conferences	67
Offered workshops or events that taught parents how to encourage gross motor activity at home	43
Other	6
Did not conduct any activities	7

a Percentages do not total 100 because programs were allowed to report more than 1 activity.

b Four programs that did not respond to the question were excluded.

c Eleven programs that did not respond to the question were excluded.

**Table 3:** Partnerships With Community Organizations to Encourage Children's Healthy Eating and Gross Motor Activity, US Head Start Programs, 2008 (N = 1,583)

Organization	% of Programs Partnering With Organization^a^
**Healthy eating (n = 1,577)^b^**	
WIC	78
USDA cooperative extension program	65
Health department	57
Food bank or pantry	38
School or school district	35
University, college, or community college	25
Grocery store	20
Community recreation department or center	15
Farmers' market	12
Faith-based organization	8
None	3
**Gross motor activity (n = 1,550)^c^**	
WIC	40
Health department	37
School or school district	32
Community recreation department or center	23
University, college, or community college	15
YMCA or YWCA	13
Faith-based organization	5
None	25

Abbreviations: WIC, Special Supplemental Nutrition Program for Women, Infants, and Children; USDA, US Department of Agriculture.

a Percentages do not total 100 because programs were allowed to report more than 1 partnership

b Six programs that did not respond to the question were excluded.

c Thirty-three programs that did not respond to the question were excluded.
